# Association between periodontitis and endometriosis: a bidirectional Mendelian randomization study

**DOI:** 10.3389/fendo.2024.1271351

**Published:** 2024-02-29

**Authors:** Bilun Jin, Pengfei Wang, Peiqi Liu, Yijie Wang, Yi Guo, Chenxu Wang, Yue Jia, Rui Zou, Shaojie Dong, Lin Niu

**Affiliations:** ^1^ Key Laboratory of Shaanxi Province for Craniofacial Precision Medicine Research, College of Stomatology, Xi’an Jiaotong University, Xi’an, China; ^2^ Clinical Research Center of Shaanxi Province for Dental and Maxillofacial Diseases, College of Stomatology, Xi’an Jiaotong University, Xi’an, China; ^3^ College of Stomatology, Xi’an Jiaotong University, Xi’an, China; ^4^ Centre of Stomatology, West China Xiamen Hospital of Sichuan University, Xiamen, China; ^5^ Bioinspired Engineering and Biomechanics Center (BEBC), Xi’an Jiaotong University, Xi’an, China

**Keywords:** causal associations, inflammation, bacterial spread, infertility, periodontal diseases

## Abstract

**Introduction:**

A potential association between periodontitis and endometriosis has been indicated in previous observational studies. Nevertheless, the causal link between these two disorders has not been clarified.

**Methods:**

Based on publicly available genome-wide association study (GWAS) summary datasets, we conducted a bidirectional Mendelian randomization (MR) study to investigate the relationship between periodontitis and endometriosis and its subtypes. Single nucleotide polymorphisms (SNPs) strongly associated with candidate exposures at the genome-wide significance level (*P* < 5 × 10^−8^) were selected as instrumental variables (IVs). The inverse variance-weighted regression (IVW) was performed to estimate the causal effect of periodontitis on endometriosis. We further conducted two sensitivity analyses, MR-Egger and weighted median, to test the validity of our findings. The main results were replicated via data from the UK Biobank. Finally, a reverse MR analysis was performed to evaluate the possibility of reverse causality.

**Results:**

The IVW method suggested that periodontitis was positively associated with endometriosis of the pelvic peritoneum (OR = 1.079, 95% CI = 1.016 to 1.146, *P* = 0.014). No causal association was indicated between periodontitis and other subtypes of endometriosis. In reversed analyses, no causal association between endometriosis or its subtypes and periodontitis was found.

**Conclusions:**

Our study provided genetic evidence on the causal relationship between periodontitis and endometriosis of the pelvic peritoneum. More studies are necessary to explore the underlying mechanisms.

## 1 Introduction

Periodontitis is a chronic inflammation characterized by loss of periodontal tissue support and alveolar bone, clinical attachment loss, and bleeding gums (1). An updated study verified that 46% of adults from the United States had periodontitis (2). The oral microbial communities detuning in periodontitis could elicit immune subversion and contribute to diseases in distance (3). Therefore, periodontitis not only affects oral functions but is also associated with some systemic diseases (4). Diseases of the cardiovascular, neurodegenerative, and even reproductive systems have been confirmed to be associated with periodontitis in various observational studies until now (5). However, limited research explored the causal effects between those diseases and periodontitis. Further study of the causality between periodontitis and associated comorbidities will provide novel insights into systemic disease treatment.

Endometriosis, similar to periodontitis, is a chronic inflammatory disease in the reproductive system. It affects 5%–10% of women of reproductive age worldwide and has been defined as a major reason for infertility (6). Endometriosis is divided into three phenotypes based on extrauterine sites where endometrial glands and stroma abnormally present 1) superficial peritoneal lesions (SUPs), 2) ovarian endometriomas (OMAs), and 3) deep infiltrating endometriosis (DIE) (7). SUP penetration is limited to 5 mm under the peritoneal surface layer (8). SUP is the least severe endometriosis with the mildest symptoms. OMA refers to chocolate cysts in the ovary, which contain a dark brown fluid (9). DIE is deemed to be the most terrible subtype with an erosive extent deeper than 5 mm under the peritoneum. Patients with DIE always suffer from severe pain (10). These phenotypes also differ at the cellular level. Single-cell profiling suggested more disorders of hormonal, inflammatory, and immunological signatures in DIE, compared with SUP and OMA (8). Because of the heterogeneity of clinical features and invasive diagnostic methods, there is an average of 6.7 years before definitive diagnosis (11, 12).

When focusing on the relationship between endometriosis and periodontitis, an earlier cross-sectional study (*N* = 2,664) based on the National Health and Nutrition Examination Survey demonstrated that women with endometriosis had a 57% higher risk of gingivitis and periodontitis than those without [adjusted odds ratio (OR) = 1.57, 95% CI = 1.06, 2.33] (13). A case–control study (*N* = 50) showed that participants with endometriosis had a higher gingival index than normal, and moderate and severe periodontitis was more common in women with endometriosis compared with normal groups (14). In a word, some observational studies have revealed a potential correlation between periodontitis and endometriosis. However, the issue of whether there exists a causal relationship between periodontitis and endometriosis or its subtypes remains unknown.

Mendelian randomization (MR) is an economical and time-saving option to explore the causal effects. Single-nucleotide polymorphisms (SNPs) are assigned randomly during the formation of a sperm cell. They could be used to reduce reverse causation or confounders and then assess the causal relationship between exposures and outcomes (15, 16). Bidirectional MR is one of the developed MR, which is performed in both ways to reduce misleading estimates from elementary MR (17). Some studies have used bidirectional MR to explore the association direction between periodontitis and different diseases, such as arthritis (18), depression (19), and psoriasis (20).

Here, we performed a bidirectional two-sample MR study between periodontitis and endometriosis. SNPs as instrumental variables (IVs) and their associations with outcomes were selected from relevant genome-wide association studies (GWASs). In addition, we selected three endometriosis subgroup datasets in order to represent SUP, OMA, and DIE, respectively. We aim to provide more genetic evidence to define the relationship between periodontitis and endometriosis.

## 2 Materials and methods

### 2.1 Study design

**Figure 1:**
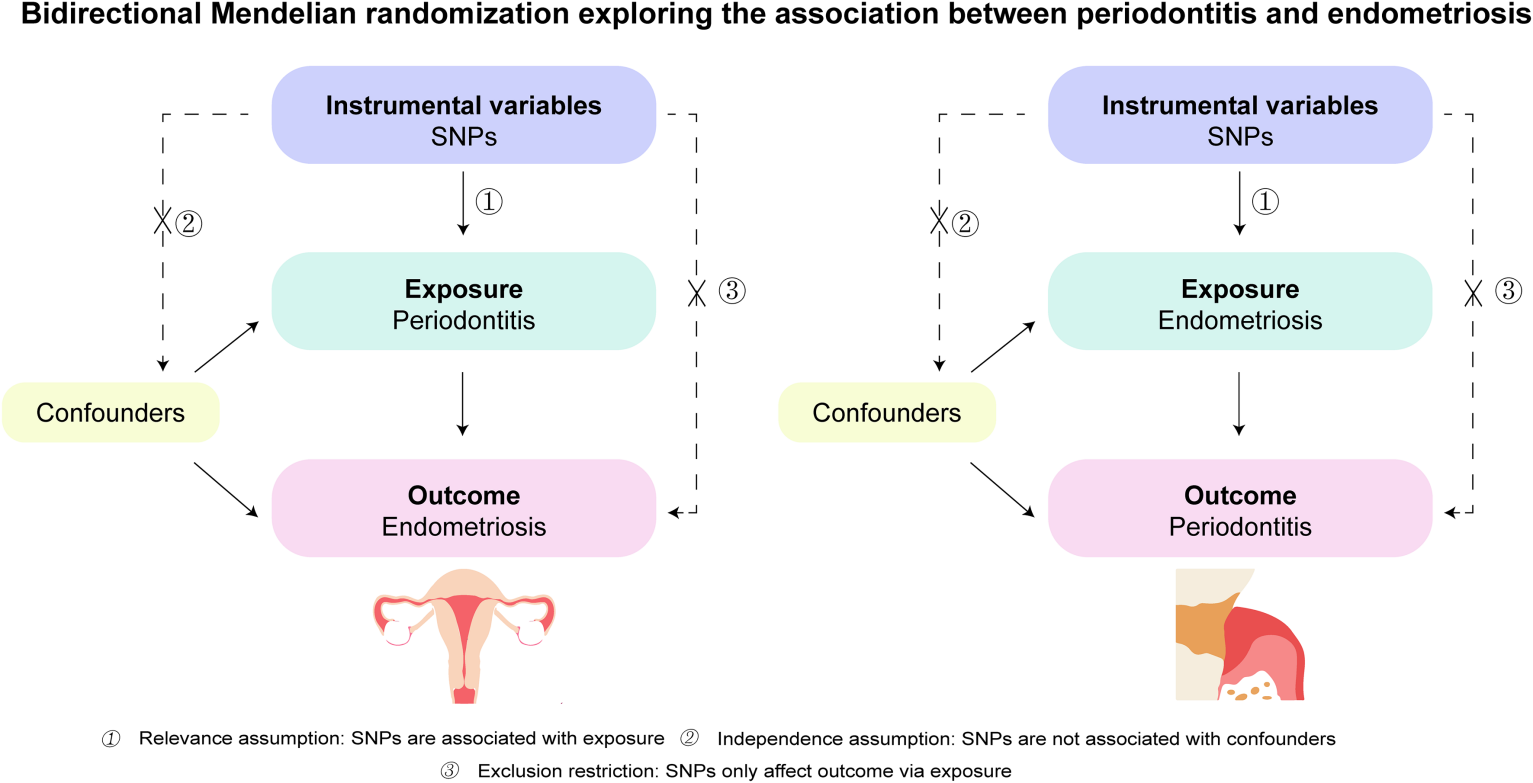
Schematics for the bidirectional Mendelian randomization design. Mendelian randomization requires valid genetic instrumental variants satisfying three assumptions. SNPs, single-nucleotide polymorphisms.

A bidirectional two-sample Mendelian randomization study was utilized to explore the causal association between periodontitis and endometriosis. We also performed a two-sample MR analysis using the dataset from the UK Biobank (UKB) to validate the causality of periodontitis on endometriosis. As shown in **Figure 1**, the study is based on three vital assumptions: firstly, there are strong associations between exposure and IVs. Secondly, IVs are not associated with the confounders. Thirdly, IVs should totally affect outcomes through exposure.

The present study was based on publicly available GWAS summary data, and ethical consent had been obtained in the original studies (21–25). All the data can be used without any restrictions.

### 2.2 GWAS datasets of periodontitis on the risk of endometriosis

We selected the IVs associated with periodontitis from three published GWAS datasets (23–25). All the participants were Europeans, including Dutch, German, and European-American. SNPs associated with the exposure at the genome-wide significance level (*P* < 5 × 10^−8^) were selected.

The datasets of endometriosis and its subgroups were obtained from FinnGen. The FinnGen study was initiated in 2017, which included more than half a million participants (age >18 years) who lived in Finland (26). The FinnGen GWAS summary data were extracted from the MRC-IEU database for endometriosis overall (8,828 cases, 68,969 controls), endometriosis of the ovary (3,231 cases, 68,969 controls), endometriosis of the pelvic peritoneum (2,953 cases, 68,969 controls), and also endometriosis of the rectovaginal septum and vagina (1,360 cases, 68,969 controls). Then, we also chose another dataset of endometriosis from UKB as the validation. UKB is a large prospective study, which aims to investigate the role of genetics, environment, and lifestyle in the causes of leading diseases. Its data come from 500,000 volunteers aged 40–69 in the United Kingdom (27). More details of exposure and outcome datasets are shown in **Supplementary Table 1**.

### 2.3 GWAS datasets of endometriosis on the risk of periodontitis

The exposure data of endometriosis and its phenotypes were obtained from the same FinnGen study as mentioned above. The outcome data for the risk of periodontitis were obtained from a GWAS meta-analysis conducted by the Gene Lifestyle Interactions in Dental Endpoints (GLIDE) on Europeans (22). Seven primary studies were included in the analysis. Periodontitis was recognized via the Centers for Disease Control and Prevention/American Academy of Periodontology definitions (28), probing depth (29), or even self-reported (30). The details of the primary studies are shown in **Supplementary Table 2**. In our study, we excluded the data of people with Hispanic/Latino ancestry. Data from European ancestry were collected from the original analysis.

### 2.4 Selection of instrumental variables

SNPs that were strongly associated with exposure at the genome-wide significance level (*P* < 5 × 10^−8^) were selected as IVs. We conducted several quality-control measures to select qualified IVs. First, the independence of SNPs was assessed based on stringent criteria (*r* ^2^ > 0.001; clumping window < 10,000 kb). Second, we used the PhenoScanner tool to check whether any of the selected SNPs were associated with potential confounders at the outcome. We set the threshold at genome-wide significance (*P* < 5 × 10^−8^) when using the PhenoScanner tool. Third, proxy SNPs were not used as IVs if SNPs were not in the 1000G reference panel. In addition, SNPs with a minor allele frequency of less than 0.01 should be excluded to avoid potential bias from the original GWAS due to the low confidence. The *R* ^2^ and *F* statistics were also calculated to avoid bias from weak instruments. SNPs were excluded if the *F* statistics were less than 10.

For the analyses of periodontitis as exposure, we selected five SNPs as valid IVs (**Supplementary Table 3**). In reversed analyses, during the selection of IVs associated with endometriosis, rs58502716 was not in the 1000G reference panel, and it was removed from the instruments. In addition, SNPs associated with endometriosis of the rectovaginal septum and vagina at a genome-wide significance level of *P* < 5 × 10^−6^ were selected in order to obtain enough IVs. rs76109112, rs139869063, rs200290589, and rs117783935 were eliminated as their *F* statistics were no more than 10. Finally, 10, 10, 5, and 13 SNPs were recognized as IVs associated with endometriosis overall, endometriosis of the ovary, endometriosis of the pelvic peritoneum, and endometriosis of the rectovaginal septum and vagina, respectively (**Supplementary Tables 4–7**).

### 2.5 Statistical analysis

The inverse variance-weighted (IVW) method in the random-effects model was applied as a dominating approach to analyze the bidirectional causal relationship between periodontitis and endometriosis. Weighted median, MR-Egger, and simple median were also added as complementary approaches to reduce potential horizontal pleiotropy and bias from the IVW method (31). The weighted median method could generate an unbiased estimate if more than half of the weight from effective IVs (32). MR-Egger is more suitable if the *P*_intercept_ *<* 0.05 because it can provide the estimates after pleiotropy is corrected (33).

To test the validity of our findings, sensitivity analyses were conducted using weighted median and MR-Egger regression. Q-tests were performed in both IVW and MR-Egger regression to assess potential heterogeneity. The MR-Egger intercept was used to assess whether the included SNPs had potential horizontal pleiotropy. Weighted median provides consistent estimates when at least 50% of the information is from valid instrumental variables. Leave-one-out analyses were also utilized to estimate the causality and heterogeneity of the study (34). As for the pleiotropy analysis, the *P*-value of the MR-Egger intercept less than 0.05 is considered to suggest the pleiotropy of the study (35).

Additionally, the statistical power of each analysis was calculated via mRnd, an online calculator (https://shiny.cnsgenomics.com/mRnd/). All data were analyzed by R (version 3.1.5), combined with the R package “TwoSampleMR.” A *P*-value < 0.05 was considered to be statistically significant. This study followed the Strengthening the Reporting of Observational Studies in Epidemiology (STROBE) guideline (**Supplementary Table 8**).

## 3 Results

### 3.1 The causal effect of periodontitis on endometriosis

**Figure 2:**
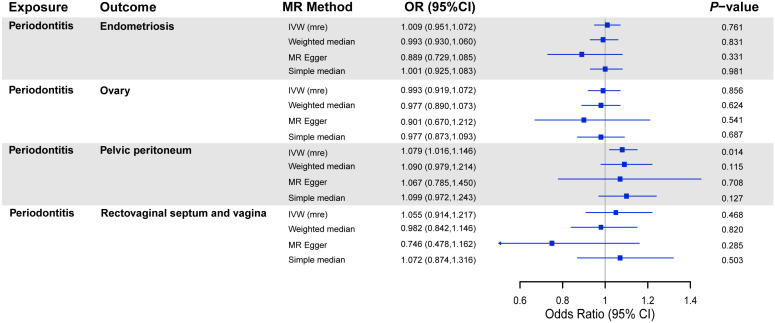
MR analyses on the association between periodontitis and risk of endometriosis. OR, odds ratio; CI, confidence interval; IVW (mre), multiplicative random effect inverse-variance weighted; SNPs, single-nucleotide polymorphisms.

The results of MR analyses on the association between periodontitis and the risk of endometriosis are shown in **Figure 2**. In the subgroup analyses, we observed that periodontitis was positively associated with endometriosis of the pelvic peritoneum (OR = 1.079, 95% CI = 1.016 to 1.146, *P* = 0.014). The replication of the main results using UKB data supported the null causality of periodontitis on endometriosis (**Supplementary Figure 1**). No causal association was indicated between periodontitis and other subtypes of endometriosis. Suggestive estimates were observed in weight median, MR-Egger, and simple median methods similarly.

Although other robust MR methods did not manifest a significant association between periodontitis and endometriosis of the pelvic peritoneum, the sensitivity analysis showed no potential horizontal pleiotropy (**Table 1**). Meanwhile, no heterogeneity existed in the IVW analyses, and none of the SNPs had a distinctive effect on estimates in the leave-one-out analyses (**Supplementary Figures 2–10**).

**Table 1:** Results of potential pleiotropy and heterogeneity assessments using different MR analyses.

Exposure	Outcome	Heterogeneity		Pleiotropy
		*P*-value for Cochran’s *Q*	Cochran’s *Q* statistic	*P*-value for MR-Egger intercept
Periodontitis	Endometriosis	0.307	4.813	0.284
Periodontitis	Endometriosis (validation)	0.434	3.799	0.232
Periodontitis	Ovary	0.476	3.510	0.553
Periodontitis	Pelvic peritoneum	0.738	1.989	0.944
Periodontitis	Rectovaginal septum and vagina	0.254	5.347	0.209
Endometriosis	Periodontitis	0.744	5.958	0.400
Ovary	Periodontitis	0.833	5.018	0.806
Pelvic peritoneum	Periodontitis	0.279	5.082	0.999
Rectovaginal septum and vagina	Periodontitis	0.048	21.144	0.763

### 3.2 The causal effect of endometriosis on periodontitis

**Figure 3:**
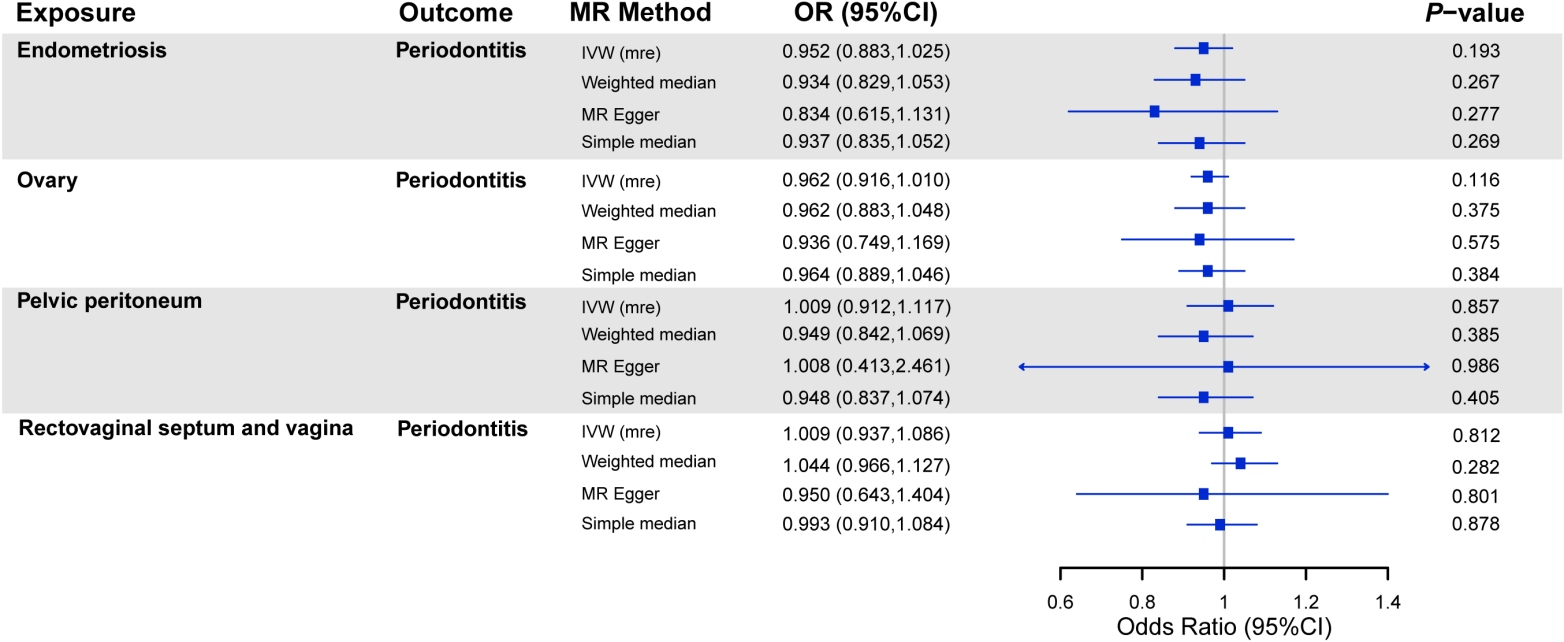
MR analyses on the association between endometriosis and risk of periodontitis. *Note*: OR, odds ratio; CI, confidence interval; IVW (mre), multiplicative random effect inverse-variance weighted; SNPs, single-nucleotide polymorphisms.

The results of the MR analyses on the association between endometriosis and the risk of periodontitis are shown in **Figure 3**. There was no causal effect of endometriosis or its subtypes on periodontitis. Consistent estimates were provided through other robust methods. As for the sensitivity analysis, no heterogeneity was found among SNPs, except for endometriosis of the rectovaginal septum and vagina (*P* = 0.048) (**Table 1**). In view of this excess heterogeneity, a random-effects analysis was performed as the main method in this study (36). Furthermore, no evidence for pleiotropy was observed via MR-Egger analyses. No pivotal difference emerged in estimates after we singly removed SNP and repeated the MR analysis.

Our study had a power of > 80% to detect an effect of OR = 1.255 of periodontitis on the risk of endometriosis and a power of > 80% to detect an effect of OR = 1.394 of endometriosis on the risk of periodontitis, on a 5% significance level.

## 4 Discussion

Periodontitis has been a momentous public health problem. It not only damages oral health but also becomes a potential cause of some systemic conditions (37). Endometriosis is a common chronic disease with several symptoms, including painful menstrual cramps, abdominal pain, and so on (38). The etiology of endometriosis is unclear, so there are still difficulties in treatment. Some observational studies reported the correlation between periodontitis and endometriosis, but the causal effect cannot be affirmed (13, 14, 39). To our knowledge, this was an innovative study to investigate the bidirectional causal effect between periodontitis and endometriosis. Our research showed a potential cause effect of periodontitis on endometriosis of the pelvic peritoneum. In reversed analyses, there was no causal association of endometriosis or its subtypes on the risk of periodontitis.

**Figure 4:**
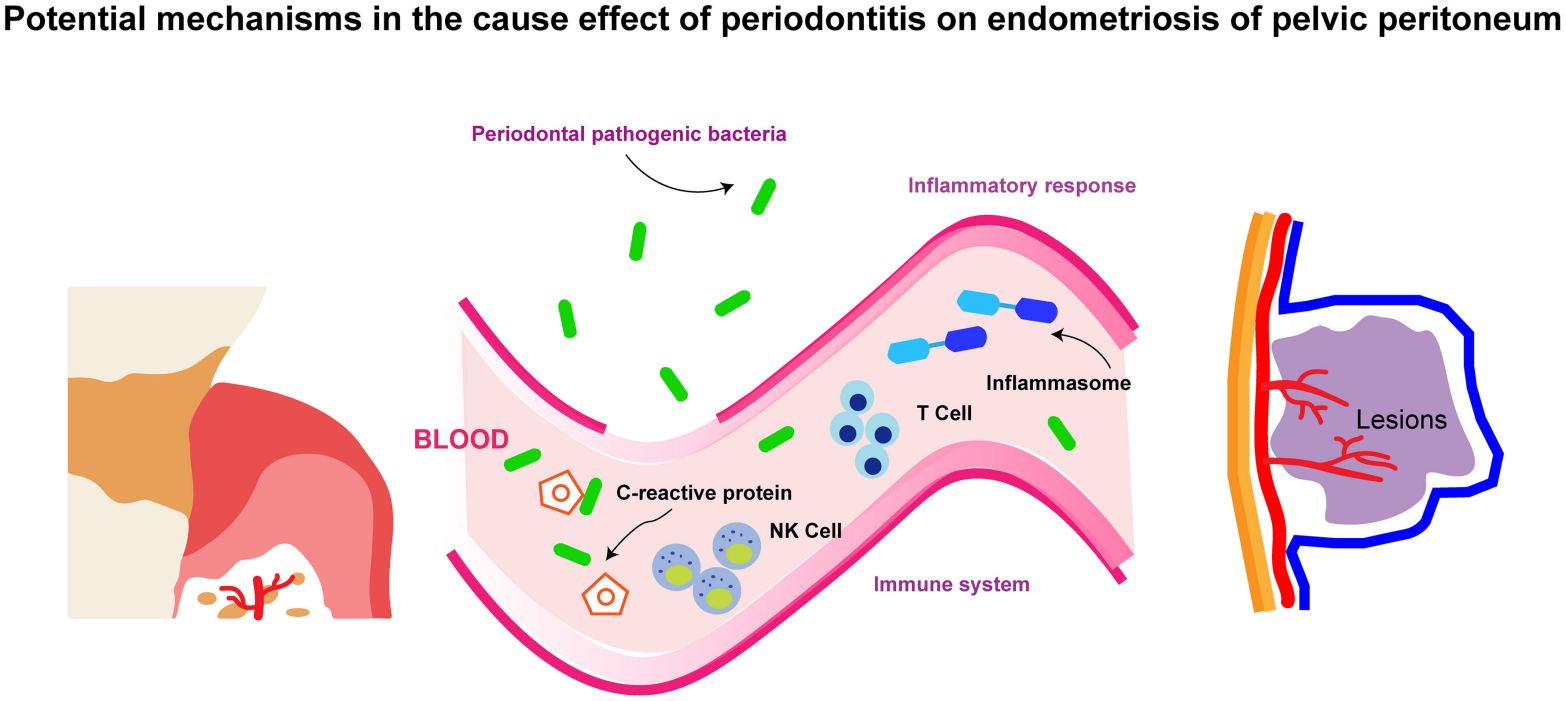
The schematic diagram for the potential mechanisms in the cause effect of periodontitis on endometriosis of pelvic peritoneum.

A promising finding in this study is the causal effect of periodontitis on endometriosis of the pelvic peritoneum, which cast new light on the pathogenesis of endometriosis. Previous research surrounding the etiology of endometriosis of the pelvic peritoneum manifested that cells from the endometrium evade immune surveillance in the peritoneum and contribute to the disease, but other mechanisms are uncertain (40). The cause effect of periodontitis on endometriosis of the pelvic peritoneum might be explained by the mechanisms provided below (and these mechanisms are also summarized in **Figure 4**):

### 4.1 Periodontal pathogenic bacteria

Oral bacteria such as *Porphyromonas gingivalis* and *Fusobacterium nucleatum* are prone to transmit into the placenta and induce intrauterine infection (41–43). Similarly, periodontal pathogens might locate in the pelvic peritoneum and then exacerbate endometriosis *in situ*. *Porphyromonas gingivalis* could activate peritoneal macrophages and trigger the production of interleukin-1 (IL-1) (44). Subsequent inflammatory cascades, together with increasing IL-1 expression, are pivotal throughout the development of endometriosis in the pelvic peritoneum (45). The frequency of *Fusobacterium* in endometriosis and endometrial tissues was significantly higher in endometriosis patients than in normal women. Animal studies also demonstrated that *Fusobacterium* from the oral cavity was a possible source of infecting the uterus through hematogenous transmission (46).

### 4.2 Inflammatory response

Periodontitis patients usually have a higher count of leukocytes, elevated levels of circulating C-reactive protein, as well as lower levels of hemoglobin and reduced numbers of red blood cells (47). Inflammasome, a multiprotein complex, is also considered as an intermedia. Periodontitis might increase Nlrp7 (a kind of inflammasome) and promote the progression of endometriosis (48). In a word, systemic inflammatory response is conjectured as a mechanism by which periodontitis causes systemic diseases.

### 4.3 Immune system

Immune dysfunction has been certified as a key cause of endometriosis of the peritoneum. For example, a genomics single-cell RNA-sequencing study first reported that T-cell receptor-positive macrophages, increasing macrophages, and natural killer (NK) dendritic cells existed in the human peritoneal fluid of endometriosis (49). NK cell therapies are also a promising treatment for endometriosis (50). In addition, reducing the immune response induced by *P. gingivalis* is helpful in alleviating some metabolic diseases associated with periodontitis (51). There is reason to suspect that periodontitis causes endometriosis through autoimmune dysregulation.

Up to now, accepted treatments for endometriosis patients include continuous medical therapies and surgeries to remove pathological tissue (52). However, persistent medications suppress hormone levels and produce side effects, such as hair loss, acne, and mood changes, and operations would lead to inevitable relapse (53). This MR study revealed periodontitis as a novel point to manage endometriosis patients. Targeting a lower risk of periodontitis may therefore improve the prognosis of people with endometriosis of the pelvic peritoneum. In other words, supportive periodontal therapies and routine oral hygiene interventions may produce beneficial effects in treating women with endometriosis of the pelvic peritoneum. In the future, some clinical randomized controlled trials with more samples should be performed to verify the causal relationship. More research is necessary not only to clarify the underlying biological pathways but also to confirm therapeutic targets for endometriosis of the pelvic peritoneum.

The bidirectional MR study has some strengths. Firstly, genetic liability plays a crucial role in the course of periodontitis and endometriosis (18, 38). GWASs allowed larger sample sizes and less bias from population structure. Prevalence rates in the database were almost consistent with those reported in the literature. Hence, MR analysis is suitable for the inference of causality between both (54). Meanwhile, the MR analyses on both sides clarified the direction of causality. Secondly, endometriosis was further subdivided into three subgroups. An interesting finding is that periodontitis might be a cause of endometriosis of the pelvic peritoneum, which has not been realized before.

Some limitations should also be considered. Firstly, IVs had a deficient association with endometriosis of the rectovaginal septum and vagina, since the threshold of *P*-value was set to 5 × 10^−6^. Therefore, more convincing evidence is needed to prove the causality between endometriosis of the rectovaginal septum and vagina and periodontitis. Secondly, a weak power was found in each analysis, due to OR close to 1, so more participants were required to detect the causal effects in the future (55).

## 5 Conclusions

In summary, this bidirectional MR study indicated a positive causal relationship between periodontitis and endometriosis of the pelvic peritoneum in the European population. More epidemiological investigations are indispensable to explore the causality between those two diseases. Further research on the mechanism is also needed in the future.

## Data availability statement

The original contributions presented in the study are included in the article/**Supplementary Material**. Further inquiries can be directed to the corresponding authors.

## Ethics statement

Ethical approval had been obtained in all original published studies. The OpenGWAS Database is a publicly available dataset, and GWAS of oral diseases complied with all relevant ethical regulations, including the Declaration of Helsinki, and ethical approval for data collection and analysis was obtained by each study from local boards.

## Author contributions

BJ: Conceptualization, Data curation, Formal analysis, Writing – original draft. PW: Data curation, Formal analysis, Investigation, Methodology, Software, Writing – review & editing. PL: Formal analysis, Investigation, Methodology, Validation, Writing – review & editing. YW: Data curation, Formal analysis, Methodology, Software, Visualization, Writing – review & editing. YG: Investigation, Methodology, Software, Writing – review & editing. CW: Investigation, Software, Visualization, Writing – review & editing. YJ: Data curation, Investigation, Methodology, Writing – review & editing. RZ: Funding acquisition, Supervision, Visualization, Writing – review & editing. SD: Funding acquisition, Investigation, Methodology, Project administration, Resources, Software, Writing – review & editing. LN: Conceptualization, Funding acquisition, Investigation, Project administration, Resources, Supervision, Validation, Writing – review & editing.
