# Supplementary material for: Association between periodontitis and endometriosis: a bidirectional Mendelian randomization study
Source: Front Endocrinol (Lausanne). 2024 Feb 29;15:1271351. doi: 10.3389/fendo.2024.1271351 (PMC10937447; doi:10.3389/fendo.2024.1271351)
Supplement: Supplementary file 1 [file DataSheet_2.docx]

Supplementary Material

# Supplementary Tables

**Table 1.** Information of exposure and outcome datasets in detail.

| Trait | GWAS | N case | N control | Ancestry |
| --- | --- | --- | --- | --- |
| Periodontitis as exposure | PMID: 19897590; DOI: 10.1093/hmg/ddp508 | 438 | 1320 | European |
|  | PMID: 28449029; DOI: 10.1093/hmg/ddx151 | 851 | 6580 | European |
|  | PMID: 30218097; DOI: 10.1038/s41431-018-0265-5 | 4924 | 7301 | European |
| Periodontitis as outcome | PMID: 31235808; DOI: 10.1038/s41467-019-10630-1 | 17353 | 28210 | European |
| Endometriosis | finn-b-N14_ENDOMETRIOSIS | 8288 | 68969 | European |
| Endometriosis (Validation) | ukb-d-N80 | 1496 | 359698 | European |
| Endometriosis of ovary | finn-b-N14_ENDOMETRIOSIS_OVARY | 3231 | 68969 | European |
| Endometriosis of pelvic peritoneum | finn-b-N14_ENDOMETRIOSIS_PELVICPERITONEUM | 2953 | 68969 | European |
| Endometriosis of rectovaginal septum and vagina | finn-b-N14_ENDOMETRIOSIS_RECTPVAGSEPT_VAGINA | 1360 | 68969 | European |

*Note:* GWAS, genome-wide association studies.

**Table 2.** The details of seven primary studies in GWAS meta-analysis conducted by the Gene Lifestyle Interactions in Dental Endpoints (GLIDE) on periodontitis.

| **Study** | **Full name** | **Age** | **Ancestral groups included in GWAS** | **Standards of periodontitis** |  |
| --- | --- | --- | --- | --- | --- |
| ARIC | Atherosclerosis Risk in Communities | 45-64 years | European | Centers for Disease Control and Prevention/American Academy of Periodontology definitions |  |
|  |  |  |  |  |  |
|  |  |  |  |  |  |
|  |  |  |  |  |  |
| COHRA1 | The Center for Oral Health in Appalachia cohort 1 (COHRA1) part of GENEVA caries | 18+years | European | The probing depth is equal or greater than 5.5 mm in 2 or more sextants |  |
|  |  |  |  |  |  |
|  |  |  |  |  |  |
|  |  |  |  |  |  |
| HCHS/SOL | Hispanic Community Health Study/Study of Latinos | 18-74 years | Hispanic/ Latino | Centers for Disease Control and Prevention/American Academy of Periodontology definitions |  |
|  |  |  |  |  |  |
|  |  |  |  |  |  |
|  |  |  |  |  |  |
| SHIP | Study of Health in Pomerania | 20-81 years | European | Centers for Disease Control and Prevention/American Academy of Periodontology definitions |  |
|  |  |  |  |  |  |
|  |  |  |  |  |  |
|  |  |  |  |  |  |
| SHIP-TREND | Study of Health in Pomerania Trend | 20-83 years | European | Centers for Disease Control and Prevention/American Academy of Periodontology definitions |  |
|  |  |  |  |  |  |
|  |  |  |  |  |  |
|  |  |  |  |  |  |
| TWINGENE | Swedish Twin Biobank | 46-93 years | European | Self-reported |  |
|  |  |  |  |  |  |
|  |  |  |  |  |  |
|  |  |  |  |  |  |
| WGHS | Women's Genome Health Study | 45+years | European | Self-reported |  |
|  |  |  |  |  |  |

**Table 3.** The characteristics of the selected SNPs on periodontitis.

| **SNP** | **Chr:pos** | **Effect allele** | **Other allele** | **EAF** | ***P*-value** | **Beta** | **SE** | **R^2^(100%)** | **F** |
| --- | --- | --- | --- | --- | --- | --- | --- | --- | --- |
| rs1537415 | 9:138529722 | C | G | 0.41 | 5.51×10^-9^ | 0.464 | 0.079 | 0.714 | 12.634 |
| rs4284742 | 19:52131733 | G | A | 0.76 | 1.34×10^-8^ | 0.293 | 0.051 | 0.264 | 19.655 |
| rs2738058 | 8:6821617 | T | C | 0.43 | 6.78×10^-10^ | 0.247 | 0.040 | 0.339 | 25.286 |
| rs16870060 | 8:104098515 | G | T | 0.91 | 3.69×10^-9^ | 0.307 | 0.052 | 0.158 | 19.305 |
| rs729876 | 16:13388778 | T | C | 0.82 | 9.77×10^-9^ | 0.215 | 0.039 | 0.211 | 25.894 |

*Note:* SNP, single-nucleotide polymorphism; Chr, chromosome; pos, position; Beta, estimate of the effect of the association; SE, standard error; R^2^, percentage of the variation of tea intake explained by the SNP; F, F-statistic.

**Table 4.** The characteristics of the selected SNPs on endometriosis.

| **SNP** | **Chr:pos** | **Effect allele** | **Other allele** | **EAF** | ***P*-value** | **Beta** | **SE** | **R^2^(100%)** | **F** |
| --- | --- | --- | --- | --- | --- | --- | --- | --- | --- |
| rs10917151 | 1:22422721 | A | G | 0.17 | 4.03×10^-13^ | 0.169 | 0.023 | 0.056 | 43.102 |
| rs6546324 | 2:67856490 | C | A | 0.69 | 4.31×10^-11^ | -0.125 | 0.019 | 0.068 | 52.630 |
| rs28517654 | 4:55993468 | C | T | 0.24 | 3.86×10^-11^ | -0.136 | 0.021 | 0.063 | 48.779 |
| rs7455028 | 6:152034386 | T | C | 0.34 | 1.13×10^-11^ | 0.125 | 0.018 | 0.070 | 54.346 |
| rs58415480 | 6:152562271 | G | C | 0.23 | 5.09×10^-26^ | 0.222 | 0.021 | 0.062 | 47.618 |
| rs140993356 | 6:151802237 | G | A | 0.06 | 1.01×10^-17^ | 0.325 | 0.038 | 0.034 | 26.385 |
| rs10757277 | 9:22124450 | G | A | 0.42 | 1.25×10^-8^ | -0.101 | 0.018 | 0.073 | 56.178 |
| rs17694933 | 9:22164309 | A | G | 0.42 | 4.71×10^-14^ | 0.133 | 0.018 | 0.073 | 56.496 |
| rs11031005 | 11:30226356 | C | T | 0.17 | 2.63×10^-23^ | -0.238 | 0.024 | 0.054 | 41.840 |
| rs9706167 | 12:95629435 | A | G | 0.15 | 7.69×10^-11^ | 0.162 | 0.025 | 0.052 | 40.160 |

*Note:* SNP, single-nucleotide polymorphism; Chr, chromosome; pos, position; Beta, estimate of the effect of the association; SE, standard error; R^2^, percentage of the variation of tea intake explained by the SNP; F, F-statistic.

**Table 5.** The characteristics of the selected SNPs on endometriosis of ovary.

| **SNP** | **Chr:pos** | **Effect allele** | **Other allele** | **EAF** | ***P*-value** | **Beta** | **SE** | **R^2^(100%)** | **F** |
| --- | --- | --- | --- | --- | --- | --- | --- | --- | --- |
| rs1209731 | 1:169324793 | C | T | 0.98 | 3.38×10^-8^ | 0.530 | 0.096 | 0.014 | 10.416 |
| rs3820687 | 1:22357229 | T | A | 0.23 | 7.11×10^-11^ | 0.205 | 0.031 | 0.044 | 31.846 |
| rs1023692 | 2:67868756 | C | T | 0.75 | 1.62×10^-8^ | -0.172 | 0.030 | 0.046 | 32.894 |
| rs12331471 | 4:56001509 | C | T | 0.25 | 4.91×10^-14^ | -0.233 | 0.031 | 0.045 | 32.257 |
| rs57689380 | 6:151734998 | C | T | 0.06 | 2.85×10^-10^ | 0.365 | 0.058 | 0.024 | 17.271 |
| rs58415480 | 6:152562271 | G | C | 0.23 | 6.50×10^-19^ | 0.283 | 0.032 | 0.043 | 31.347 |
| rs12524009 | 6:19731384 | C | T | 0.15 | 2.51×10^-8^ | 0.206 | 0.037 | 0.038 | 27.100 |
| rs851984 | 6:152023191 | A | G | 0.44 | 2.21×10^-9^ | 0.158 | 0.026 | 0.052 | 37.878 |
| rs17694933 | 9:22164309 | A | G | 0.42 | 2.19×10^-10^ | 0.168 | 0.027 | 0.052 | 37.735 |
| rs11031005 | 11:30226356 | C | T | 0.17 | 1.94×10^-14^ | -0.274 | 0.036 | 0.039 | 27.932 |

*Note:* SNP, single-nucleotide polymorphism; Chr, chromosome; pos, position; Beta, estimate of the effect of the association; SE, standard error; R^2^, percentage of the variation of tea intake explained by the SNP; F, F-statistic.

**Table 6.** The characteristics of the selected SNPs on endometriosis of pelvic peritoneum.

| **SNP** | **Chr:pos** | **Effect allele** | **Other allele** | **EAF** | ***P*-value** | **Beta** | **SE** | **R^2^(100%)** | **F** |
| --- | --- | --- | --- | --- | --- | --- | --- | --- | --- |
| rs61778046 | 1:22358789 | T | G | 0.19 | 5.83×10^-12^ | 0.243 | 0.035 | 0.039 | 28.300 |
| rs1551642 | 4:55994210 | C | T | 0.27 | 2.32×10^-9^ | -0.187 | 0.031 | 0.045 | 32.019 |
| rs1971256 | 6:151816011 | C | T | 0.22 | 3.58×10^-11^ | 0.222 | 0.034 | 0.041 | 29.821 |
| rs10122243 | 9:22158924 | T | C | 0.41 | 2.26×10^-10^ | 0.177 | 0.028 | 0.050 | 35.806 |
| rs11031005 | 11:30226356 | C | T | 0.17 | 1.10×10^-10^ | -0.240 | 0.037 | 0.037 | 26.855 |

*Note:* SNP, single-nucleotide polymorphism; Chr, chromosome; pos, position; Beta, estimate of the effect of the association; SE, standard error; R^2^, percentage of the variation of tea intake explained by the SNP; F, F-statistic.

**Table 7.** The characteristics of the selected SNPs on endometriosis of rectovaginal septum and vagina.

| **SNP** | **Chr:pos** | **Effect allele** | **Other allele** | **EAF** | ***P*-value** | **Beta** | **SE** | **R^2^(100%)** | **F** |
| --- | --- | --- | --- | --- | --- | --- | --- | --- | --- |
| rs4669753 | 2:11728080 | A | G | 0.38 | 4.75×10^-7^ | -0.207 | 0.041 | 0.035 | 24.330 |
| rs10170312 | 2:232489859 | C | T | 0.36 | 4.46×10^-6^ | 0.191 | 0.042 | 0.034 | 24.038 |
| rs1441948 | 3:77384466 | A | G | 0.72 | 3.98×10^-6^ | 0.206 | 0.045 | 0.032 | 22.371 |
| rs62305883 | 4:101963570 | C | T | 0.18 | 1.82×10^-6^ | -0.254 | 0.053 | 0.027 | 18.832 |
| rs17007348 | 4:120817221 | A | C | 0.06 | 2.08×10^-6^ | 0.390 | 0.082 | 0.017 | 12.180 |
| rs3765282 | 6:6654517 | A | C | 0.15 | 1.64×10^-6^ | 0.268 | 0.056 | 0.025 | 17.857 |
| rs113470730 | 6:152014260 | C | T | 0.05 | 3.04×10^-8^ | 0.527 | 0.095 | 0.015 | 10.515 |
| rs17062448 | 6:102197319 | C | G | 0.11 | 2.14×10^-6^ | 0.307 | 0.065 | 0.022 | 15.456 |
| rs7759516 | 6:151838245 | C | T | 0.22 | 1.36×10^-9^ | 0.299 | 0.049 | 0.029 | 20.283 |
| rs11031005 | 11:30226356 | C | T | 0.17 | 5.39×10^-7^ | -0.268 | 0.054 | 0.027 | 18.691 |
| rs2987324 | 13:67759573 | A | T | 0.38 | 4.08×10^-6^ | 0.190 | 0.041 | 0.034 | 24.212 |
| rs2073284 | 17:76117401 | G | A | 0.52 | 3.61×10^-6^ | -0.187 | 0.040 | 0.035 | 24.813 |
| rs2967683 | 19:8785951 | C | T | 0.27 | 9.75×10^-8^ | 0.241 | 0.045 | 0.031 | 22.074 |

*Note:* SNP, single-nucleotide polymorphism; Chr, chromosome; pos, position; Beta, estimate of the effect of the association; SE, standard error; R^2^, percentage of the variation of endometriosis of rectovaginal septum and vagina explained by the SNP; F, F-statistic.

| **Table 8.** STROBE-MR Checklist. | | |  |  |
| --- | --- | --- | --- | --- |
| Item No. | Section | Checklist item | Manuscript section and paragraph |  |
| **Title and abstract** | | | |  |
| 1 | Title and abstract | Indicate mendelian randomization (MR) as the study’s design in the title and/or the abstract if that is a main purpose of the study. | Title and abstract |  |
| **Introduction** | | | |  |
| 2 | Background | Explain the scientific background and rationale for the reported study. What is the exposure? Is a potential causal relationship between exposure and outcome plausible? Justify why MR is a helpful method to address the study question. | Introduction, paragraphs 1-4 |  |
| 3 | Objectives | State specific objectives clearly, including prespecified causal hypotheses (if any). State that MR is a method that, under specific assumptions, intends to estimate causal effects. | Introduction, paragraph 3 |  |
| **Methods** | | | |  |
| 4 | Study design and data sources | Present key elements of the study design early in the article. Consider including a table listing sources of data for all phases of the study. For each data source contributing to the analysis, describe the following: | Materials and methods; Figure 1 |  |
|  | a | Setting: Describe the study design and the underlying population, if possible. Describe the setting, locations, and relevant dates, including periods of recruitment, exposure, follow-up, and data collection, when available. | Materials and methods, "Study design" section |  |
|  | b | Participants: Report the eligibility criteria and the sources and methods of selection of participants. Report the sample size and whether any power or sample size calculations were carried out prior to the main analysis. | Materials and methods, " Instrumental variables selection" and "Statistical analysis" sections; Table S1 |  |
|  | c | Describe measurement, quality control, and selection of genetic variants. | Materials and methods, " Instrumental variables selection" section |  |
|  | d | For each exposure, outcome, and other relevant variables, describe methods of assessment and diagnostic criteria for diseases. | Materials and methods, " Instrumental variables selection" section |  |
|  | e | Provide details of ethics committee approval and participant informed consent, if relevant. | Materials and methods, "Study design" section |  |
| 5 | Assumptions | Explicitly state the 3-core instrumental variable (IV) assumptions for the main analysis (relevance, independence, and exclusion restriction), as well assumptions for any additional or sensitivity analysis. | Materials and methods, "Study design" and "Statistical analysis" sections; Figure 1 |  |
| 6 | Statistical methods: main analysis | Describe statistical methods and statistics used. | Materials and methods, "Statistical analysis" sections |  |
|  | a | Describe how quantitative variables were handled in the analyses (ie, scale, units, model). | Materials and methods, "Statistical analysis" sections |  |
|  | b | Describe how genetic variants were handled in the analyses and, if applicable, how their weights were selected. | Materials and methods, "Statistical analysis" sections |  |
|  | c | Describe the MR estimator (eg, 2-stage least squares, Wald ratio) and related statistics. Detail the included covariates and, in case of 2-sample MR, whether the same covariate set was used for adjustment in the 2 samples. | Materials and methods, "Statistical analysis" sections |  |
|  | d | Explain how missing data were addressed. | N/A |  |
|  | e | If applicable, indicate how multiple testing was addressed. | N/A |  |
| 7 | Assessment of assumptions | Describe any methods or prior knowledge used to assess the assumptions or justify their validity. | Materials and methods, "Statistical analysis" sections |  |
| 8 | Sensitivity analyses and additional analyses | Describe any sensitivity analyses or additional analyses performed (eg, comparison of effect estimates from different approaches, independent replication, bias analytic techniques, validation of instruments, simulations). | Materials and methods, "Statistical analysis" sections |  |
| 9 | Software and preregistration |  |  |  |
|  | a | Name statistical software and package(s), including version and settings used. | Materials and methods, "Statistical analysis" sections |  |
|  | b | State whether the study protocol and details were preregistered (as well as when and where). | N/A |  |
| **Results** | | | |  |
| 10 | Descriptive data |  |  |  |
|  | a | Report the numbers of individuals at each stage of included studies and reasons for exclusion. Consider use of a flow diagram. | Table S1 |  |
|  | b | Report summary statistics for phenotypic exposure(s), outcome(s), and other relevant variables (eg, means, SDs, proportions). | Figure 2, 3 |  |
|  | c | If the data sources include meta-analyses of previous studies, provide the assessments of heterogeneity across these studies. | N/A |  |
|  | d | For 2-sample MR: i. Provide justification of the similarity of the genetic variant–exposure associations between the exposure and outcome samples. ii. Provide information on the number of individuals who overlap between the exposure and outcome studies. | N/A |  |
| 11 | Main results |  |  |  |
|  | a | Report the associations between genetic variant and exposure and between genetic variant and outcome, preferably on an interpretable scale. | Table S2-6 |  |
|  | b | Report MR estimates of the relationship between exposure and outcome and the measures of uncertainty from the MR analysis, on an interpretable scale, such as odds ratio or relative risk per SD difference. | Results, , paragraph 1-3 |  |
|  | c | If relevant, consider translating estimates of relative risk into absolute risk for a meaningful time period. | N/A |  |
|  | d | Consider plots to visualize results (eg, forest plot, scatterplot of associations between genetic variants and outcome vs between genetic variants and exposure). | Figure S1-8 |  |
| 12 | Assessment of assumptions |  |  |  |
|  | a | Report the assessment of the validity of the assumptions. | Results, paragraph 1-3 |  |
|  | b | Report any additional statistics (eg, assessments of heterogeneity across genetic variants, such as I2, Q statistic, or E-value). | Results, paragraph 4 |  |
| 13 | Sensitivity analyses and additional analyses |  |  |  |
|  | a | Report any sensitivity analyses to assess the robustness of the main results to violations of the assumptions. | Results, paragraph 1-3 |  |
|  | b | Report results from other sensitivity analyses or additional analyses. | Results, paragraph 2,3 |  |
|  | c | Report any assessment of the direction of the causal relationship (eg, bidirectional MR). | N/A |  |
|  | d | When relevant, report and compare with estimates from non-MR analyses. | N/A |  |
|  | e | Consider additional plots to visualize results (eg, leave-one-out analyses). | Results, Figure S1-S8 |  |
| **Discussion** | | | |  |
| 14 | Key results | Summarize key results with reference to study objectives. | Discussion, paragraph 1 |  |
| 15 | Limitations | Discuss limitations of the study, taking into account the validity of the IV assumptions, other sources of potential bias, and imprecision. Discuss both direction and magnitude of any potential bias and any efforts to address them. | Discussion, paragraph 5 |  |
| 16 | Interpretation |  |  |  |
|  | a | Meaning: Give a cautious overall interpretation of results in the context of their limitations and in comparison with other studies. | Discussion, paragraph 2-4 |  |
|  | b | Mechanism: Discuss underlying biological mechanisms that could drive a potential causal relationship between the investigated exposure and the outcome, and whether the gene-environment equivalence assumption is reasonable. Use causal language carefully, clarifying that IV estimates may provide causal effects only under certain assumptions. | Discussion, paragraph 2-4 |  |
|  | c | Clinical relevance: Discuss whether the results have clinical or public policy relevance, and to what extent they inform effect sizes of possible interventions. | Discussion, paragraph 4,5 |  |
| 17 | Generalizability | Discuss the generalizability of the study results (a) to other populations, (b) across other exposure periods/timings, and (c) across other levels of exposure. | Discussion, paragraph 5 |  |
| **Other Information** | | | |  |
| 18 | Funding | Describe sources of funding and the role of funders in the present study and, if applicable, sources of funding for the databases and original study or studies on which the present study is based. | Fundings section |  |
| 19 | Data and data sharing | Provide the data used to perform all analyses or report where and how the data can be accessed, and reference these sources in the article. Provide the statistical code needed to reproduce the results in the article or report whether the code is publicly accessible and, if so, where. | Data availability statement section |  |
| 20 | Conflicts of interest | All authors should declare all potential conflicts of interest. | Conflicts of interest section |  |
| Reference: Skrivankova, V.W., Richmond, R.C., Woolf, B.A.R., Yarmolinsky, J., Davies, N.M., Swanson, S.A., et al. (2021). Strengthening the Reporting of Observational Studies in Epidemiology Using Mendelian Randomization: The STROBE-MR Statement. JAMA 326, 1614-1621. doi: 10.1001/jama.2021.18236 | | | |  |
|  |  |  |  |  |
|  |  |  |  |  |

# Supplementary Figures


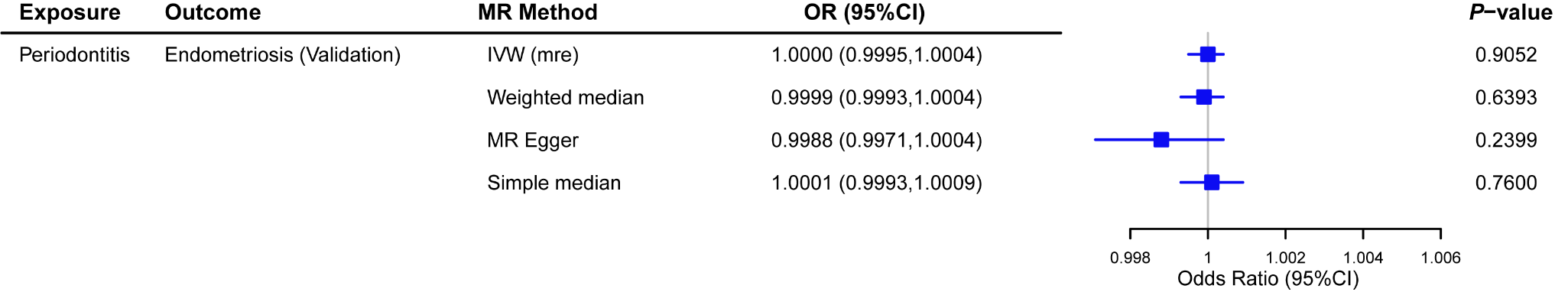


**Figure S1.** The results of MR analyses on the association between periodontitis and the risk of endometriosis (Validation).


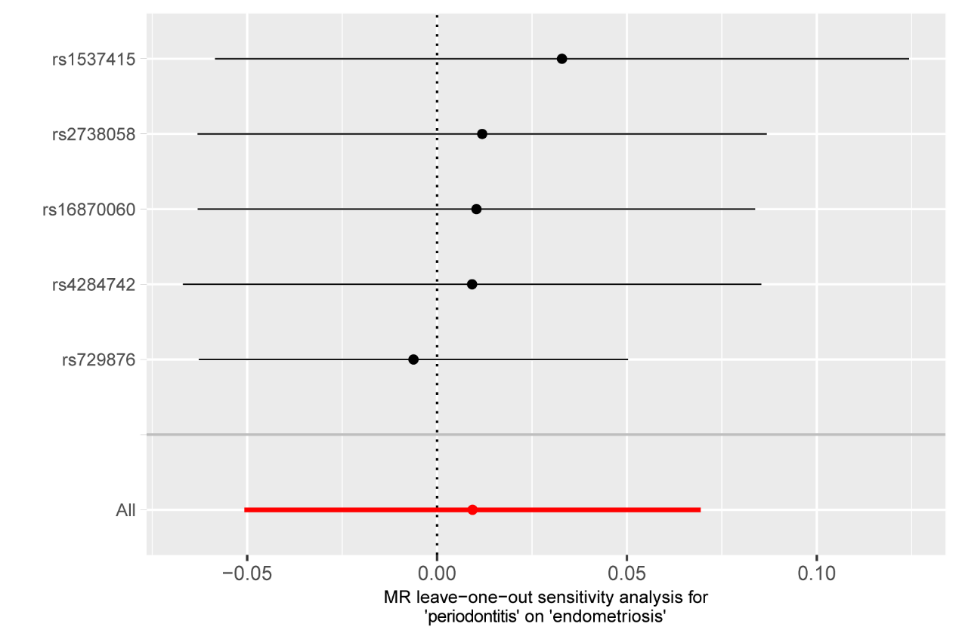


**Figure S2.** Leave-one-out analysis of individual and combined SNP MR estimates for periodontitis on risk of endometriosis.


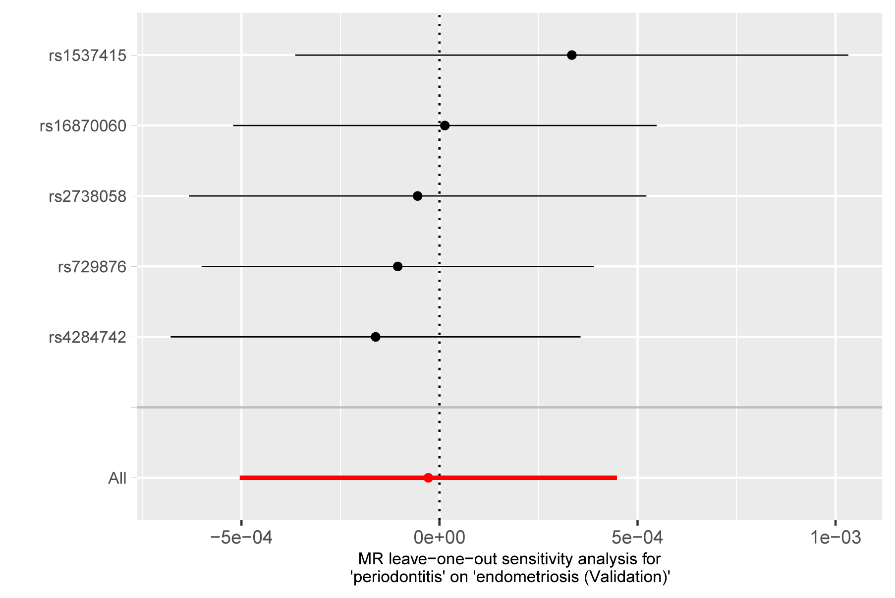


**Figure S3.** Leave-one-out analysis of individual and combined SNP MR estimates for periodontitis on risk of endometriosis (Validation).


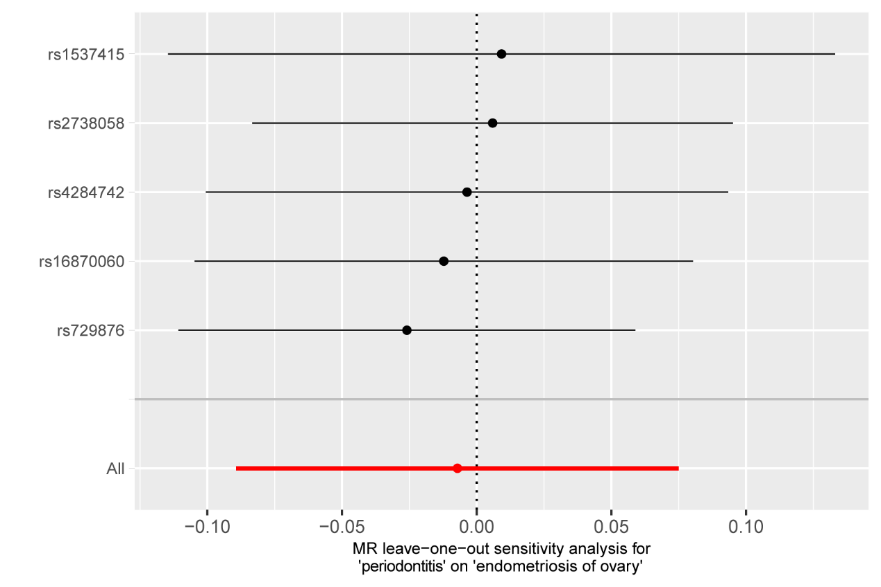


**Figure S4.** Leave-one-out analysis of individual and combined SNP MR estimates for periodontitis on risk of endometriosis of ovary.


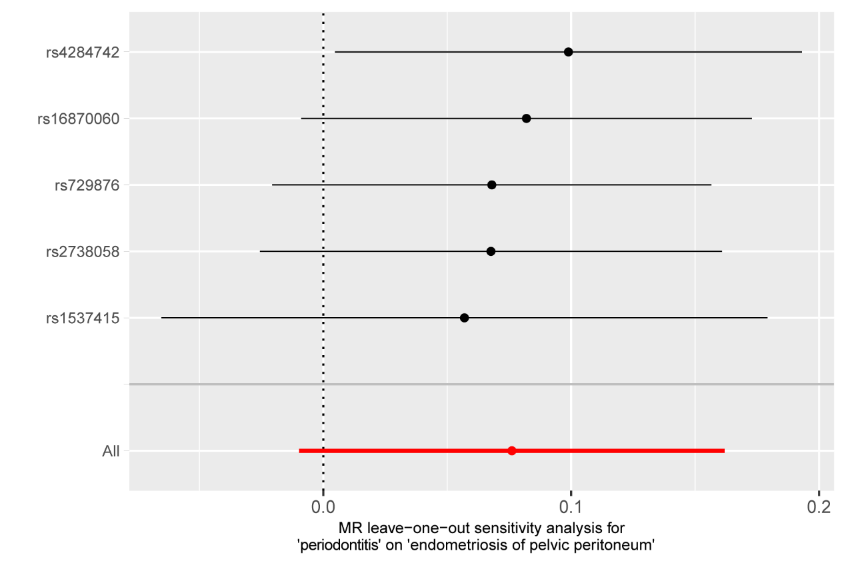


**Figure S5.** Leave-one-out analysis of individual and combined SNP MR estimates for periodontitis on risk of endometriosis of pelvic peritoneum.


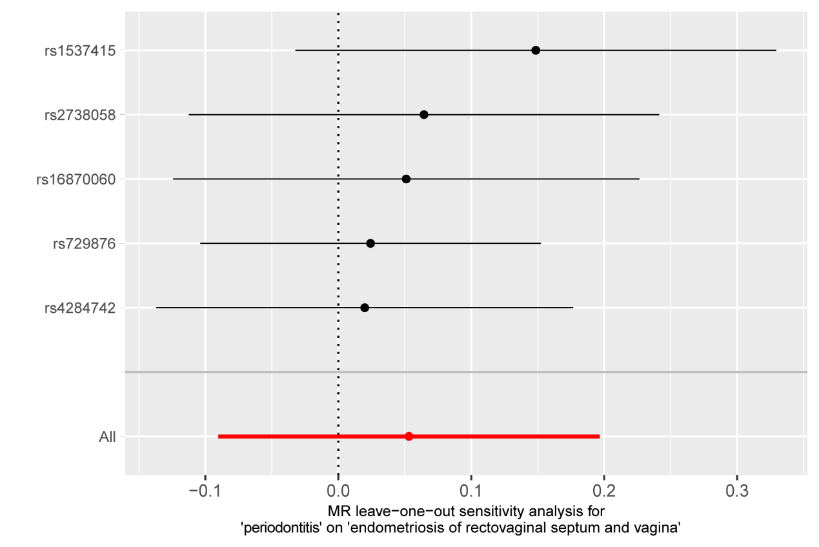


**Figure S6.** Leave-one-out analysis of individual and combined SNP MR estimates for periodontitis on risk of endometriosis of rectovaginal septum and vagina.


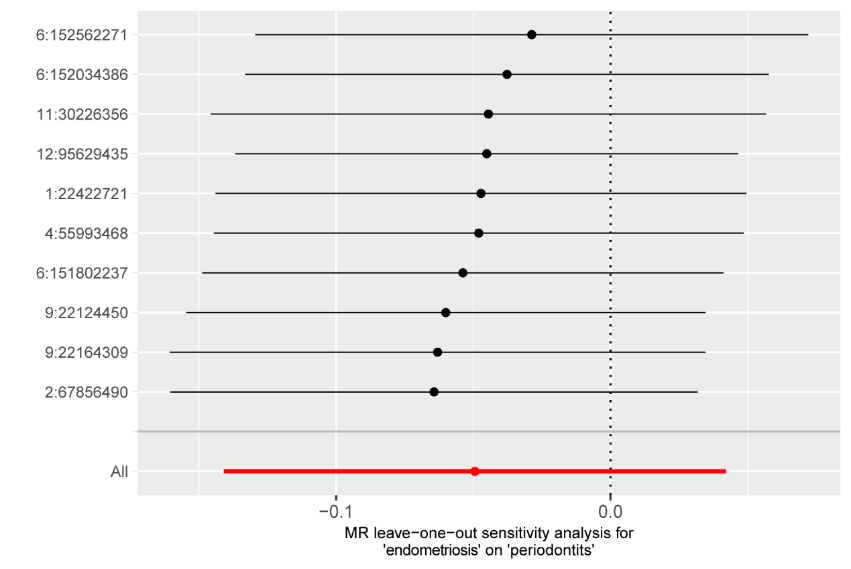


**Figure S7.** Leave-one-out analysis of individual and combined SNP MR estimates for endometriosis on risk of periodontitis.


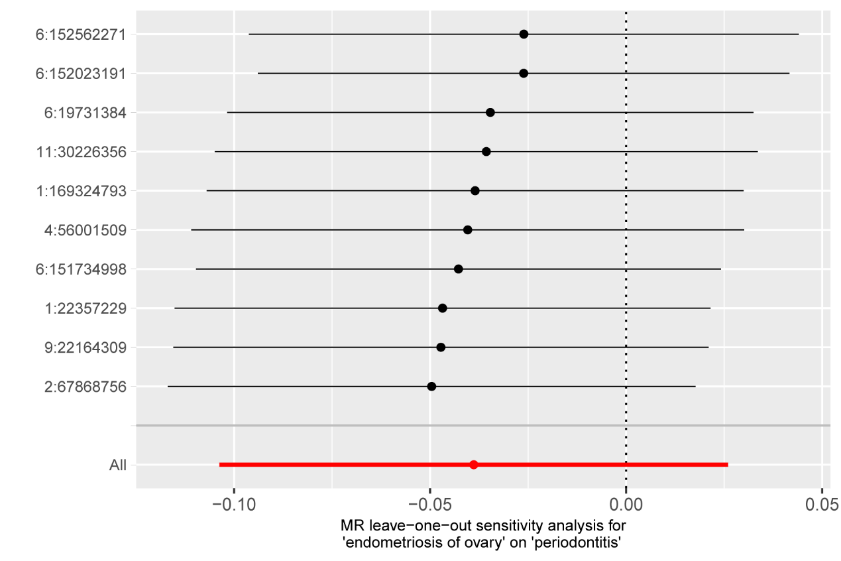


**Figure S8.** Leave-one-out analysis of individual and combined SNP MR estimates for endometriosis of ovary on risk of periodontitis.


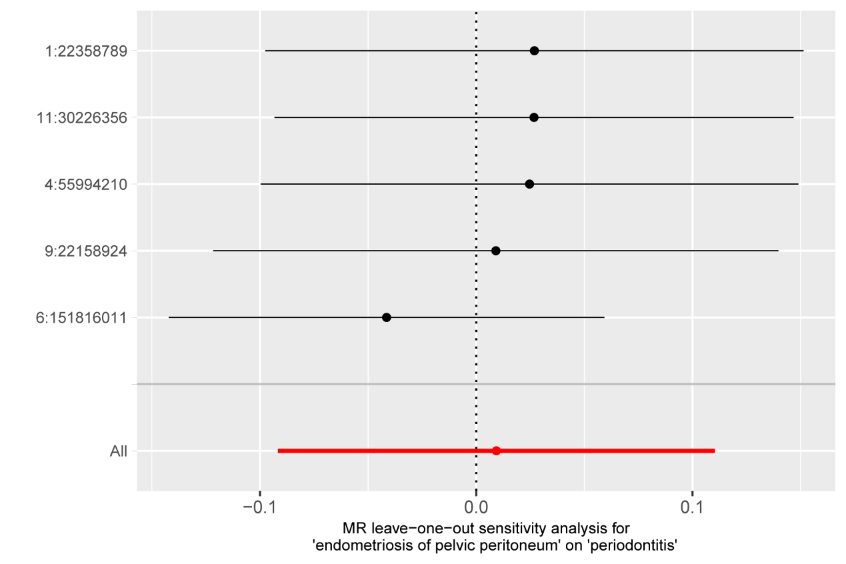


**Figure S9.** Leave-one-out analysis of individual and combined SNP MR estimates for endometriosis of pelvic peritoneum on risk of periodontitis.


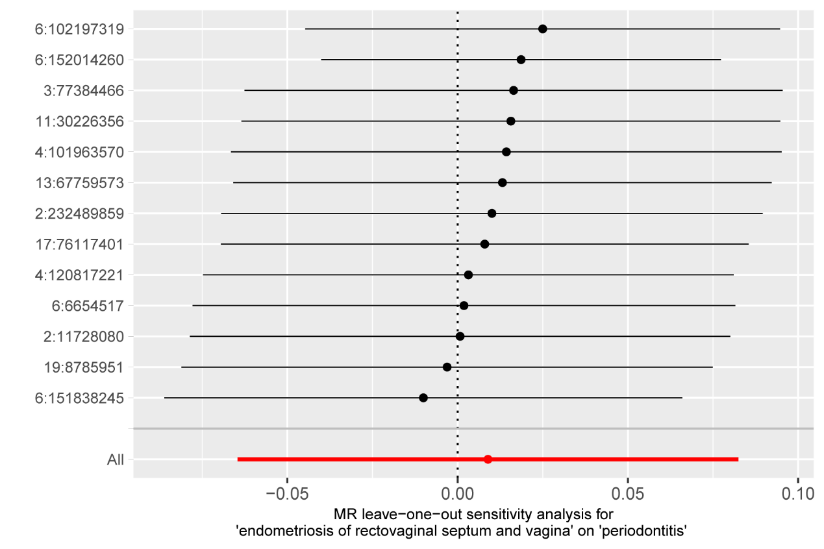


**Figure S10.** Leave-one-out analysis of individual and combined SNP MR estimates for endometriosis of rectovaginal septum and vagina on risk of periodontitis.
